# Value-based radiology: what is the ESR doing, and what should we do in the future?

**DOI:** 10.1186/s13244-021-01056-9

**Published:** 2021-07-27

**Authors:** European Society of Radiology (ESR), Adrian P. Brady, Jacob Visser, Guy Frija, Núria Bargalló, Andrea Rockall, Boris Brkljacic, Michael Fuchsjäger, Judy Birch, Minerva Becker, Thomas Kröncke

**Affiliations:** European Society of Radiology, Am Gestade 1, 1010 Vienna, Austria

**Keywords:** Value-based radiology, Patients, Communication, Education, Clinical decision support

## Abstract

Value-based radiology (VBR) is rapidly gaining ground as a means of considering the input of radiology practice into individual and societal healthcare, and represents a welcome move away from older metrics focused on counting studies performed, without consideration of whether these studies contributed positively to patient management or to society as a whole. Intrinsic to the process of considering whether radiology activity confers value is recognising the breadth of involvement of radiology in healthcare delivery; previous ESR and multi-society publications have explored this, and have sought to highlight the many ways in which our specialty contributes to patient welfare. This paper is intended to highlight some current ESR activities which already contribute substantially to value creation and delivery, and to outline a selection of practical steps which could be taken by the ESR in the future to enhance value.

**Patient summary**

Value-based radiology (VBR) is a conceptual means of looking at the benefits conferred on patients and on society as a whole by provision of radiology services, as opposed to older means of counting numbers of radiology studies performed, without consideration of whether or not those studies contributed overall value. VBR will become increasingly important in the future as a means of determining resources. The ESR has been a leader in advancing VBR concepts and educating radiologists about this novel way of looking at what we do. This paper is designed to highlight current ESR activities which contribute value to healthcare, and to consider other ways in which the ESR could potentially support value enhancement in the future.

## Keypoints

- Value-based healthcare is a concept which is growing in influence within modern medicine.
- Radiology must adapt to value-based healthcare oncepts.
- The ESR is already engaged in many activities which add value to radiology practice. Some of these are outlined in this paper, and a series of actions is suggested which could be taken by the ESR in the future to promote value-based radiology.

## Introduction

In 2017, the European Society of Radiology (ESR) published a concept paper on Value-Based Radiology [1], outlining the ideas behind value-based healthcare, and considering how these might relate to and influence the practice of radiology. In 2020, as part of a multi-society group involving the ESR, the American College of Radiology (ACR), the Canadian Association of Radiologists (CAR), the Radiological Society of North America (RSNA), the Royal Australian & New Zealand College of Radiologists (RANZCR) and the International Society for Strategic Studies in Radiology (IS3R), we published a short paper in the Journal of the American Medical Association (JAMA) [2] and a longer multi-society statement in multiple journals [3], further developing the discussion about how radiology provides value in healthcare, and how this value could be enhanced. In early 2021, we also published the results from a survey of patients in 22 countries, which explored what patients consider to be the most-valuable aspects of radiology services they receive [4].

Traditionally, radiology output has been measured in terms of numbers, reflecting clinical productivity (numbers of reports generated or procedures performed), academic productivity (numbers of publications, academic lectures etc.) and financial performance (fees earned, costs incurred), with some attention additionally given to patient satisfaction and compliance with regulations [5]. Value-Based Radiology, as a general concept, seeks to change the focus from a numerical assessment of what we do to one that takes account of the contribution of our work to patient outcomes and societal benefit [1–3].

In this paper, the ESR now seeks to establish the many activities the society supports and engages in which contribute value to patient healthcare, and, secondly, to identify practical steps which can be taken now, or for which we can advocate, to enhance value creation and delivery.

### Current ESR value-adding activities

As a society primarily dedicated to radiology education, the ESR (and its predecessor organisations, the European Association of Radiology and the stand-alone European Congress of Radiology) fundamentally exists to promote good radiology practice, to provide education to our members to enhance their professional careers, and to establish and maintain standards of radiology education and service delivery across all our member countries. At the simplest level, educating radiologists to do their jobs to the best of their abilities, for the benefit of patients and society as a whole, is the vital foundation for value delivery by radiology.

Many other more-focused ESR activities also contribute value, including (but not confined to) the following:

1. **Eurosafe imaging** sets standards for dosimetry and radiation protection, provides educational modules on radiation protection, and encourages safe radiology practices, while maximising diagnostic yield [6].
2. The **iGuide Clinical Decision Support Tool** guides referrers to help them choose the most-appropriate radiological investigation to answer their particular clinical question, with the greatest efficiency and safety [7].
3. The **Esperanto Clinical Audit Tool** instructs users in the process of clinical audit, and provides audit templates to assist radiology departments begin their own processes of clinical audit, designed to strive towards continual improvement in the quality of radiology practices [8].
4. **European Commission projects**. The ESR is currently engaged in (or has recently completed) a number of important projects funded by the European Commission, designed to improve quality and patient safety. These include EUCLID (designed to define dose reference levels for CT) [9], QuADRANT (designed to assess and support clinical audit) [10] & EU-JUST-CT (designed to improve justification of CT radiation dose) [11].
5. The **Patient Advisory Group (PAG)** of the ESR meets regularly, contributes to our educational endeavours, and provides the patient viewpoint in all our activities relating to standards and quality (including participating in the production of this paper). All ESR white papers and published standards now contain summaries for patients, which provide a *precis* of the main messages of the paper in plain language for lay readers [12].
6. **Multi-disciplinary team activity**. One of the key areas where radiologists can influence patient management and outcome (beyond our core work of interpretation of studies and performance of related procedures) is by participation in multidisciplinary teams in our hospitals, being part of the specialist groups which make the key management recommendations, with access to all relevant information. The ESR strives to support this multidisciplinary role by incorporation of multidisciplinary sessions in our educational courses and congresses, making the views and needs of other specialists available to our members.
7. Publication of three peer-reviewed **journals** (*European Radiology, Insights into Imaging and European Radiology Experimental)* that report on and support advances in the field of radiology, including research, evidence-based practice guidelines and educational reviews.

## What practical actions can we now take to enhance value delivery by radiology?

1. **Referral information.** Much of what we do as radiologists is about provision and dissemination of information. Yet we are often forced to do our work in a condition of relative information starvation. The amount of clinical information provided to us when studies are requested is not always what we might wish, and key elements of important clinical data, which might help us to be more specific and definitive in our reporting, are often absent from referrals [13–15]. There are many possible reasons for this, including time constraints for the referrer, referrers for specific studies having only limited awareness of chronic conditions which may not form part of their particular relationship with the patient, delegation of the task of submitting requests for imaging studies to staff members other than the referring physician, and (paradoxically) the increasing focus on exam appropriateness, which may lead referrers to submit the minimum amount of information required to justify a request, in the belief that additional information provided may lead to a request being considered inappropriate [14]. As we move towards provision of radiology reports in a structured manner [16], we should also try to encourage provision of structured information in requests for radiology studies. This could be achieved by defining key fields of information which should be completed by referrers before requests would be accepted by PACS/RIS systems [14]. Adoption of Computerized Physician Order Entry (CPOE) systems should be encouraged. These developments would require engagement with relevant industry partners. Work would need to be done to convince referrers of the need to perform what some may see as additional work, but ensuring provision of relevant clinical information in all cases would increase the specificity and usefulness of radiology reports, with benefits for all.
2. **Electronic Medical Records.** An associated possible action relates to making all relevant clinical information available to reporting radiologists; by this we mean information not ordinarily contained within referrals, but which could influence the content of a radiology report. In effect, this would require real-time access for reporting radiologists to electronic medical records (EMRs) of patients. In most instances, assuming provision of appropriate information in the study request, accessing the EMR would not be necessary (indeed, given workload and staffing constraints in many countries, it would not be possible for radiologists to devote time to this). However, in certain complex cases, being directly able to access specific pieces of information might make all the difference between issuing a vague, indeterminate report and one that is precise, specific and relevant. Key to this would be the adoption of EMRs in as many clinical environments as possible. At present, few countries have moved entirely to the EMR environment. Estonia is one notable example of a country where every patient has an online electronic health record, amended as required via Blockchain to ensure data integrity [17]. Reliance on EMR access to obtain information necessary for accurate radiological interpretation is not appropriate in most circumstances, and would entail disadvantages: getting quick access to all information in an EMR relevant for a specific circumstance can be difficult and time-consuming, and over-reliance on the EMR for transmission of critical specific information would carry the risk of reducing the responsibility of the referrer to provide clear, accurate and comprehensive referrals. However, a summary table of main diagnoses and the current problem in the EMR could potentially ease the burden of extensive communication, if this information sits in a single, easy-to access position accessible to all. This might allow the avoidance of the need to repeatedly explain the background information, on top of the current indication. Regardless of availability of EMRs to reporting radiologists, workload and time constraints are likely to result in unselected records being accessed relatively rarely by radiologists [18]. A 2009 study from Philadelphia suggested that much greater use would be made of systems that targeted specifically-relevant patient information to be automatically displayed with imaging studies, and that this would have a significant impact on interpretation quality [18]. This ties in with Point 1 above, and is a development that could and should be advocated for, with efforts made to specify the specific information required for specific types of studies. The ESR should support endeavours to use the ever-expanding capabilities of information technology and AI to speed up access to and transfer of relevant clinical information between referrers and radiologists.
3. **Justification, decision support and protocols.** Many ESR activities are already directed towards these essential elements of ensuring the safety and quality of our work (see above) [7, 11]. The ESR should continue to encourage and support research to fill in gaps of understanding about aspects of these issues, such as differences in understanding of the responsibility for justification in different jurisdictions [19], the relative use of CDS and the proportion of appropriate examinations in public vs. private imaging institutions, and practices relating to individual protocol determination for imaging studies and the impact of these.
4. **Direct patient communication.** The working environment for radiologists has changed considerably in recent years, with many drivers combining to distance us from the patients we serve [20]. Simultaneously, patient advocates are increasingly asking for more direct access to radiologists to discuss their symptoms, imaging findings and studies. In the context of an inexorable increase in demand for what we do, not always matched by the resources needed, it may seem that the desire to engage more directly with patients is incompatible with the need to cope with our workload. We must avoid accepting this assumption. In a recently-published study, Gutzeit et al. have demonstrated that brief communication with patients following MR studies led to changes in radiologists’ reports in 42.6% of cases, and that those changes were of high clinical relevance in 32.7% [15]. A Dutch survey of negative patient reviews of radiologists published in May 2021 found that the dominant reasons for patient dissatisfaction related to poor communication by radiologists during patient engagements, or apparent uncaring attitudes shown by radiologists [21]. In our education and advocacy efforts, we must emphasise that radiologists are also clinicians, and that our direct clinical engagement with patients can have beneficial significance substantially beyond the provision of a report of an imaging study. Studies such as Gutzeit’s can be used to justify demand for sufficient resources (time, staff etc.) to allow us engage directly with patients, for their benefit. Healthcare managers must be educated to understand that benefits to patients and society can flow from accepting that this engagement should be part of a radiologist’s workload, and provided for in schedules and workplans. A practical step that can be taken by the ESR is to expand its educational provision to include courses and educational content on patient communication skills specifically tailored to the radiology environment. Formal communication training is used very effectively in settings where difficult news needs to be communicated, such as in oncology or trauma practice. This may be of great practical benefit in radiology, for improving communication within a time-pressured setting.
5. **Patient information resources.** Working with the sub-speciality groups, patient groups, allied professionals and the national societies, ESR should advocate for trusted on-line sites for patient information concerning ‘what to expect’ from different imaging procedures. In addition resources on radiation risks, in particular in paediatric imaging, should be made available [6]. Developing such a resource, with clear explanations, diagrams and frequently-asked questions (FAQs) could provide essential information to patients prior to attending for examinations, and thereby improve the overall experience of the patient while improving radiologist-patient interactions due to better patient understanding of the procedure.
6. **Imaging data sharing and protection.** The 2009 paper referenced in item 2 above identified one priority desired by radiologists as being that imaging studies and reports from outside institutions be available for review at the time of reporting new studies [18]. This is a logical goal; direct comparison with prior studies is often key to determining the significance of abnormal findings. Many countries are moving towards patient imaging studies being available across multiple sites or regional imaging networks, either through use of central archives [22], web-based archiving or patients holding portable copies of their studies. A corollary of this is the need for secure data backups and protection, as evidenced by the May 2021 ransomware attack on the Irish healthcare system IT infrastructure, which resulted in major disruption to provision of radiology services to the entire country [23]. The ESR should support endeavours to improve wide availability of patients’ imaging archives wherever possible, and to define necessary security requirements.
7. **Follow-up recommendations.** Ensuring appropriate follow-up of reported abnormal or equivocal radiological findings is ultimately the responsibility of the referrer. Nonetheless, as radiologists who generate reports containing such recommendations, we have a role in supporting timely and complete compliance with them. The more steps there are in any process, the more scope there is for inadvertent failure. Development of automatic reminder systems to alert referrers and reporting radiologists in a timely fashion to follow-up studies (or to the need to arrange follow-up) can assist in closing investigatory loops, and ensuring no patient is lost to follow-up [18]. This does not mean the burden of responsibility for further investigation (where necessary) should shift from the referrer to the radiologist. Nonetheless, the more failsafe mechanisms that exist to ensure all involved share responsibility for patient welfare, the more certain we can be that patient care is optimized, and potential value is not lost. Again, the ESR can and should support development of IT-based systems identifying and highlighting actionable items within radiology reports.
8. **Countering commoditization.** Many factors are driving increasing commoditization of radiology at present, reducing the visibility of radiologists as significant providers of healthcare, and major contributors to patient outcomes [14, 20]. Perception of what we do as simply an investigative output is inaccurate and incomplete, doing a disservice to patients and to radiologists [2, 3]. Key performance indicators (KPIs) used for imaging departments frequently focus on productivity, and rarely on quality, safety and (more difficult to define) value contributions. The ESR has attempted to define appropriate quality-based KPIs for radiation protection [24]. Similarly, we should strive to define meaningful KPIs which would more-accurately demonstrate the breadth of our contributions to healthcare, beyond study or report numbers. Furthermore, we, as radiologists, must do what we can to ensure we are perceived and understood to be full partners in clinical care of patients. In person participation of radiologists in clinical rounds, regular multidisciplinary conferences and tumour board type discussions should be developed and encouraged. In addition, encouraging radiologists to lead innovative imaging research that is clinically relevant should be supported and encouraged as an important element of raising the visibility and value of radiologists in treatment planning.
9. **Study nomenclature standardization.** Standardization of study nomenclature (especially CT), to facilitate dose comparisons between institutions and jurisdictions, and compliance with DRLs, is among the quality measures mandated under US federal legislation considered by the American College of Radiology (ACR) to be relevant for radiology [5]. The ESR can and should have a role in developing and promoting such standardized study labelling. This could be done by focussing initially on selected, achievable indications, based on specific clinical scenarios, as promoted in the EUCLID study [9].
10. **Communication with referrers.** A radiologist making a timely, accurate diagnosis may believe that he/she is contributing value to patient care, but unless that diagnosis is communicated efficiently to the referrer, and acted upon, the contribution of radiology is diminished [5]. In addition to the recommendation in item 3 above relating to communication with patients, the ESR should advocate for greater education of radiologists in matters of inter-professional communication, and for development, resourcing and implementation of IT systems for rapid communication of urgent findings. Structured reporting may significantly enhance completeness and accuracy of radiology reports [16]. The ESR should facilitate usage of Common Data Elements [25] and encourage vendors to enable usage of structured reporting templates. This would also facilitate potential automatic delivery of data to data registries for AI application development.
11. **Peer review.** Radiological error and discrepancy are inevitable, but all possible efforts should be made to minimise significant error [26]. One potential tool to achieve this is formal mandated submission of a percentage of every radiologist’s randomly-selected reports for second reading and peer review. Such systems are already in use in some countries [27]. Although peer review adds somewhat to radiologist workload, with associated impact on staff resourcing, the ESR should advocate for its wider use, and the necessary resources to make it work, in the interests of patient safety and increased value. A key component of such peer review is establishment of an open and blame-free framework allowing radiologists share and learn from cases where error or discrepancy has occurred, without fear of sanction or legal consequences from such sharing [25, 27]. The ESR could encourage adoption of such practices (and the necessary legal protections and supports) across our member countries.
12. **Artificial intelligence (AI)**. The number of software as medical devices (SaMDs) being approved for medical use by regulatory agencies is growing rapidly, particularly in medical imaging [28]. Many claims have been made for the value of machine learning algorithms in enhancing radiology diagnostic performance, and the possibility of AI applications replacing traditional radiologist activity in many areas has been prominently discussed in both academic and popular media. Despite this, there is a paucity of research demonstrating unbiased improved performance of AI over human radiologists in full diagnostic activity (as opposed to radiology sub-tasks, such as lung nodule detection), and a recent review showed little evidence of improved clinician diagnostic performance when using machine learning-based clinical decision support systems (CDSSs), with a high incidence of human clinicians over-riding the CDSS output [28]. There is no doubt that AI, in many forms, will become an increasing part of medical (including radiological) life, but what form that part will take is still unclear [29]. The ESR should support activities, such as direct AI-human data challenges, which are designed to provide verifiable validation of any benefits of AI in real-world performance. Other AI applications have the potential to have significant beneficial effects on workflow; these should be supported and highlighted for early adoption, where appropriate.
13. **Integrated diagnostics.** As stated in point 1 above, much of the work of the radiologist involves information provision and dissemination. This is also true for pathologists. Concordance workflows between those 2 could be a first step in closer integration of these specialties, with emphasis on radiologic-pathologic correlation and cross-fertilization of relevant findings. AI applications could accelerate this, with future potential for merging radiology and pathology departments into single entities, creating unified diagnostic information resources, managing information extracted from radiology and pathology reports in the clinical context of the patient [30].

## Conclusion

The process of defining, evaluating and enhancing value in the delivery of radiology services to patients and referrers should be never-ending and iterative. We can always do better, and we always be seeking means to improve. Initiatives to increase value today should, if successful, become standards tomorrow, and new value-creation objectives will continue to emerge, be defined, and, we hope, be achieved. The ESR has been a leader in value-based radiology, and already supports many endeavours aimed at increasing value. This paper attempts to identify other possible actions which could increase the value of radiology practices, and which could usefully be explored or supported by the Society. This list is neither intended to be proscriptive nor to be exhaustive. Some of the ideas discussed may not prove feasible or valuable, and many other value-creating strategies will emerge as value-based radiology continues to increase in acceptance and importance. Nonetheless, we hope that the concepts we discuss in this paper will encourage future research and developments, to continually enhance the value provided by radiology to patients and to society.

## Data Availability

All data is included in this manuscript.
